# Extra-cellular release and blood diffusion of BART viral micro-RNAs produced by EBV-infected nasopharyngeal carcinoma cells

**DOI:** 10.1186/1743-422X-7-271

**Published:** 2010-10-15

**Authors:** Claire Gourzones, Aurore Gelin, Izabela Bombik, Jihène Klibi, Benjamin Vérillaud, Joël Guigay, Philippe Lang, Stéphane Témam, Véronique Schneider, Corinne Amiel, Sonia Baconnais, Anne-Sophie Jimenez, Pierre Busson

**Affiliations:** 1 Univ Paris-sud 11, CNRS-UMR 8126 and Institut de Cancérologie Gustave Roussy, 39 rue Camille Desmoulins, F-94805 Villejuif, France; 2 Univ Pierre et Marie Curie-Paris 6, Laboratoire de Virologie, Hôpital Tenon, 4 rue de la Chine, F-75020 Paris, France; 3 Univ Paris-sud 11 and Institut de Cancérologie Gustave Roussy, cervico-facial surgery unit, 39 rue Camille Desmoulins, F-94805 Villejuif, France; 4 Service de Radiothérapie, Hôpital de la Salpêtrière, 47 bd de l'Hôpital, F-75013 Paris, France

## Abstract

**Background:**

Nasopharyngeal carcinoma (NPC) is a human epithelial malignancy consistently associated with the Epstein-Barr virus. The viral genome is contained in the nuclei of all malignant cells with abundant transcription of a family of viral microRNAs called BART miRNAs. MicroRNAs are well known intra-cellular regulatory elements of gene expression. In addition, they are often exported in the extra-cellular space and sometimes transferred in recipient cells distinct from the producer cells. Extra-cellular transport of the microRNAs is facilitated by various processes including association with protective proteins and packaging in secreted nanovesicles called exosomes. Presence of microRNAS produced by malignant cells has been reported in the blood and saliva of tumor-bearing patients, especially patients diagnosed with glioblastoma or ovarian carcinoma. In this context, it was decided to investigate extra-cellular release of BART miRNAs by NPC cells and their possible detection in the blood of NPC patients. To address this question, we investigated by quantitative RT-PCR the status of 5 microRNAs from the BART family in exosomes released by NPC cells *in vitro *as well as in plasma samples from NPC xenografted nude mice and NPC patients.

**Results:**

We report that the BART miRNAs are released in the extra-cellular space by NPC cells being associated, at least to a large extent, with secreted exosomes. They are detected with a good selectivity in plasma samples from NPC xenografted nude mice as well as NPC patients.

**Conclusions:**

Viral BART miRNAs are secreted by NPC cells *in vitro *and *in vivo*. They have enough stability to diffuse from the tumor site to the peripheral blood. This study provides a basis to explore their potential as a source of novel tumor biomarkers and their possible role in communications between malignant and non-malignant cells.

## Background

Nasopharyngeal carcinoma (NPC) is one of the most frequent virus-related malignancies in humans, following liver carcinomas associated to HBV and HCV and cervix carcinoma associated to HPV. This epithelial malignancy arises from the epithelium lining the upper part of the pharynx behind the nasal cavities. NPC incidence is variable depending on the geographic area [1]. It occurs at a very high incidence in Southern China, especially in the Guangdong and Guangxi provinces (25 cases/100 000/year) whereas it is at a low incidence in Europe or North America (about 1 case/100 000/year). There are areas of intermediate incidence whose extension has long been underappreciated and which include vast regions of South East Asia (Indonesia, Vietnam, Philippines) and North Africa (Tunisia, Algeria, Morocco) (4 to 8 cases/100 000/year). Incidence is rising in some places in Europe because of large numbers of incoming overseas immigrants. Although EBV is not the unique etiological factor of NPC, it has a role in tumor development in combination with dietary factors (consumption of traditional preserved food) and genetic predisposition [2]. Regardless of patient geographic origin, the EBV genome is contained in the nuclei of all malignant cells in virtually all NPCs (except a very small fringe of differentiated squamous tumors in Europe and North America). Most viral genes are silent but some of them are consistently expressed including genes encoding for two clusters of microRNAs called BART miRNAs [3,4].

MicroRNAs are double strand RNAs of short size (20 to 25 nt) which result from maturation of large primary transcripts and have important regulatory functions in gene expression. When they are incorporated to a multimolecular complex called RISC, they have the power to interact with target mRNAs inducing their degradation or slowing their translation [5]. Initial studies on microRNAs have been mainly focused on their functions inside the producer cells. Recently, it has been shown that microRNAs are often released in the extra-cellular medium. Moreover, they can enter cells distinct from producing cells and modify gene expression in recipient cells [6-8]. Extra-cellular transport of microRNAs is facilitated by various processes such as association with protective proteins or packaging in exosomes [9,10]. Exosomes are nanovesicles of 50 to 100 nm in diameter which are derived from the late endosomal compartment and secreted by most eukaryotic cell types [11]. Exosomes behave as extra-cellular carriers of microRNAs that they can deliver to recipient cells *in vitro *and probably also *in vivo *[7,8]. Detection of tumor microRNAs has been reported in the plasma of tumor-bearing patients for example patients affected by glioblastoma and ovarian carcinomas [12,13].

Three clusters of viral microRNAs encoded by the EBV genome have been identified in the past years [3]. One of them maps to the Bam H1 H open reading frame 1 (BHRF1) of the viral genome and is therefore called the BHRF1 cluster. The two others map to the Bam H1 A region. They are derived from primary RNAs called BARTs because they are transcribed rightward from an ORF of the Bam H1 A region (Bam H1 A rightward transcripts) [14]. Each BART cluster derives from a distinct pair of introns of the BART primary transcripts: introns 1 and 2 (cluster 1 - coordinates 138480 - 140558) and introns 3 and 4 (cluster 2 - coordinates 146334 - 149581)[14]. BHRF1 miRNAs are abundant in some EBV-infected lymphoid cell lines but they are absent or scarce in NPC cells. In contrast, BART primary transcripts and microRNAs are extremely abundant in NPC cells [4,14-16]. So far, however, it is not known whether the BART microRNAs (BART miRNAs) are secreted by NPC cells and whether they can be detected in the plasma and body fluids of NPC patients.

The aim of this study was to investigate secretion of BART miRNAs by NPC cells and their diffusion in the plasma of NPC-xenografted mice and NPC patients. We demonstrate that BART miRNAs are secreted by NPC cells *in vitro *in association with exosomes (at least a fraction of them). Moreover BART miRNAs are detected in the plasma of NPC-xenografted mice or NPC patients, thus appearing as a potential source of novel tumor biomarkers.

## Results

### Detection of BART miRNAs in xenografted NPC tumors

**Figure 1:**
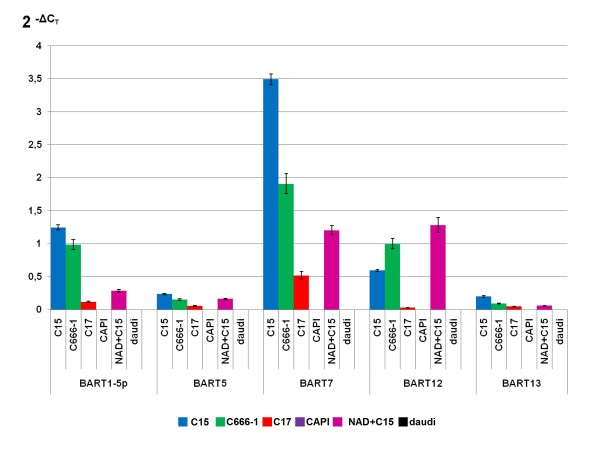
**Detection of the BART miRNAs in total RNAs extracted from NPC xenografts and EBV-infected B-cells**. Presence of BART miRNAs - miR-BART1-5p and 5 (cluster 1) and miR-BART 7-3p, 12 and 13 (cluster 2) - was assessed by real time PCR following multiplex RT-PCR. Abundance of each microRNA is assessed by 2^-ΔCT ^calculation using the small cellular RNA RNU 44 as an endogenous reference. C15, C17 and C666-1 are NPC xenografts. CAPI is a xenografted EBV-negative epithelial tumor derived from a carcinoma of unknown primary. NAD+C15 is a lymphoblastoid cell line latently infected by an EBV isolate derived from the C15 NPC xenograft. Daudi is a Burkitt lymphoma cell line naturally infected by EBV and carrying its own distinct isolate. These data are representative of two similar experiments.

Expression of a panel of 5 BART miRNAs was investigated in total RNA extracted from the C15, C17 and C666-1 NPC xenografts by quantitative PCR following multiplexed reverse-transcription (RT). Reverse transcription was performed on a multiplex mode using a set of primers specific for all 5 BART miRNAs, followed by single-mode PCR using one universal primer and one primer specific for each BART miRNA. The small non-coding RNA RNU44 was used as an endogenous reference. Our panel of BART miRNAs included members of cluster 1 (miR-BART 1-5p and 5) and cluster 2 (miR-BART 7-3p, 12 and 13). On the basis of previous publications, these microRNAs were expected to be among the most abundant BART miRNAs produced by NPC cells [4,14,15,17,18]. As anticipated, they were readily amplified from the RNA of NPC xenografts. The RNA extracted from the CAPI tumor, an EBV-negative non-NPC epithelial xenografted tumor was used as a negative control (Figure 1). In order to allow comparative analysis of BART miRNAs in NPC and EBV-infected lymphoid cells, total RNAs from 2 lymphoid cell lines were processed using the same primers and experimental conditions. Daudi was derived from a Burkitt lymphoma and carries its own EBV isolate. NAD+C15 is an LCL (lymphoblastoid cell line) derived from normal B-cells *in vitro *transformed by artificial infection using the C15 EBV isolate [4,19]. As previously reported, no BART miRNA was detected in Daudi [4]. In contrast, all 5 BART miRNAs of our panel were detected in the NAD+C15 LCL with a profile somehow similar to the C15 NPC xenograft profile (Figure 1). It is noteworthy that in NPC tumors as well as in the NAD+C15 LCL, miR-BART 7-3p was expressed at a higher level than the 4 other BART miRNAs.

### Detection of BART miRNAs in exosomes released by NPC cells *in vitro*

**Figure 2:**
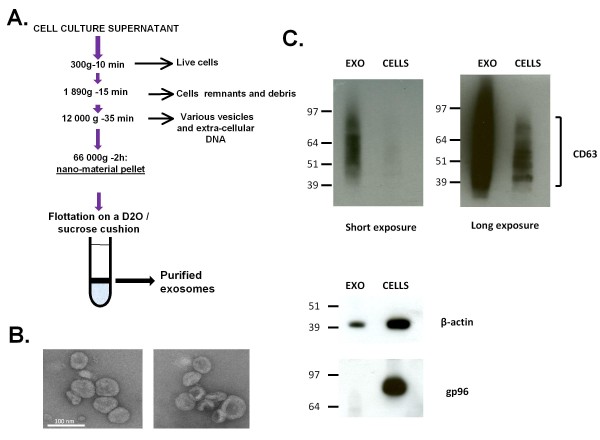
**Isolation of NPC exosomes from cell culture supernatants and quality control of exosome preparations**. **A)** Summary of the experimental procedure used for exosome purification. **B) **Negative staining electron microscopy of exosomes purified from NAD+C15 conditioned culture medium. Scale bar: 100 nm. Exosomes are characterized by a diameter of 50 to 100 nm and a frequent plate-like morphology. **C) **Western blot analysis of CD63 and gp96 in whole cell (CELLS) and exosome (EXO) protein extracts (NAD+C15). Regardless of the cell background, the CD63 tetraspanin is generally very abundant in exosomes. In contrast gp96 which is a cytoplasmic membrane protein is absent or at a very low concentration. Staining with anti-β-actin was used for loading control (although it is less abundant in exosomes than in whole cell extracts). Overall these data confirm the good quality of our exosome preparations.

**Figure 3:**
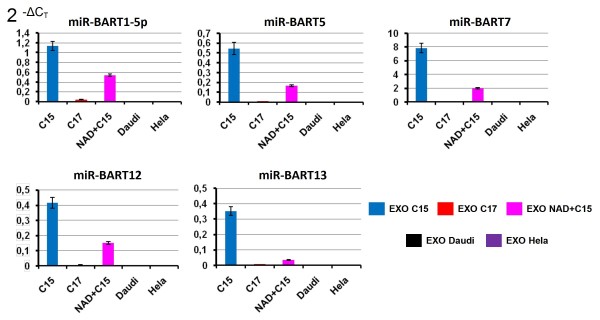
**Detection of the BART miRNAs secreted by NPC cells in association with exosomes**. Presence of 5 BART miRNAs - miR-BART1-5p and 5 (cluster 1) and miR-BART 7-3p, 12 and 13 (cluster 2) - was assessed by real time PCR following multiplex RT. Each BART miRNA is relatively abundant in the exosomes from C15 NPC cells and to a lesser extent from NAD+C15 LCL cells. The same BART miRNAs are barely detectable in C17 exosomes. As expected the BART miRNAs are absent in exosomes from Hela cells which are EBV-negative. Their absence in the exosomes from Daudi cells is consistent with their absence in Daudi cellular RNA (see Figure 1). Note that the 2^-ΔCT ^index for miR-BART 7-3p is several times higher than for other BART microRNAs. These data are representative of two similar experiments.

Several reports have shown that at least a fraction of extra-cellular microRNAs are secreted in association with exosomes [7,12,20]. Therefore, we undertook to investigate the distribution of BART miRNAs in exosomes released by malignant NPC cells. Epithelial cells from the C15 and C17 NPC xenografts were dispersed by collagenase treatment and incubated *in vitro *for 48 h in order to produce conditioned culture media. Exosomes were prepared from these conditioned media as explained in Figure 2A. Simultaneously, exosomes were prepared from permanently propagated cell lines: the NAD+C15 LCL, Daudi and Hela cells. Quality of these exosome preparations was assessed using ordinary morphological and biochemical criteria. Round vesicles of 50 to 100 nm in diameter often with a plate-like shape were observed under electron microscopy. High concentrations of the CD63 tetraspanin were obtained in the exosome extracts constrasting with the absence of the gp96 cytoplasmic protein (Figure 2B)[21]. The distribution of miR-BART 1-5p, 5, 7-3p, 12 and 13 was investigated in total RNA extracted from these exosomes using multiplexed RT combined to real-time PCR. The cellular miR-21 which is abundant in most types of human malignant cells was used as an endogenous control [22]. The highest relative concentrations of BART miRNAs were detected in exosomes released by the C15 NPC cells, followed by exosomes from the NAD+C15 LCL (Figure 3). Lower but still significant amounts of BART miRNAs were detected in exosomes from C17 NPC cells. In contrast, no BART miRNA were detected in exosomes from Daudi and Hela cells. Like for tumor RNAs, miR-BART 7-3p was more abundant than other BART miRNAs in all exosome RNA preparations.

### Detection of BART miRNAs in the plasma of xenografted NPC- bearing mice

**Figure 4:**
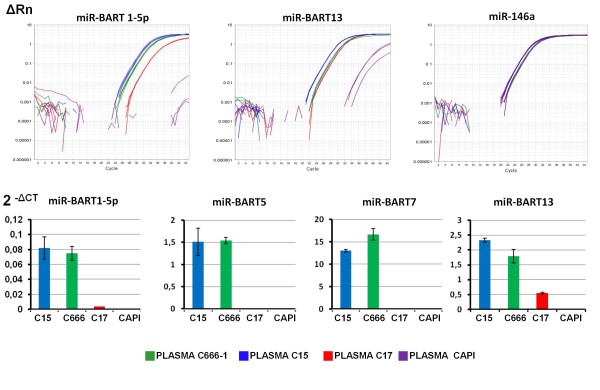
**Detection of EBV BART miRNAs in plasma samples of mice carrying xenografted NPC tumors (C15, C17, C666-1)**. Presence of 4 BART miRNAs - miR-BART1-5p and 5 (cluster 1) and miR-BART 7-3p and 13 (cluster 2) - was assessed by real time PCR following multiplex RT. Plasma samples from mice xenografted with an EBV-negative epithelial tumor (CAPI) were used as negative controls. For each type of xenografted tumor**, **PCR analysis was performed on pools of plasma samples collected from 3 or 4 mice. The cellular miR-146a which is known to be detectable in blood plasma was used as an endogenous reference [23]. **Upper panel: **amplification plots obtained for miR-BART1-5p and 13 and for mir-146a. ΔRn stands for the magnitude of the fluorescence signal generated during the PCR at each time point (with background correction). **Lower panel: **histograms presenting the 2^-ΔCT ^values for miR-BART 1-5p, 5, 7-3p and 13. All 4 BART miRNAs are relatively abundant in plasma samples from mice xenografted with C15 and C666-1 whereas they are at a low level in samples from C17 mice. This is consistent with data obtained from the corresponding tumor and cellular RNAs (see Figure 1). Like for tumor and exosome RNAs, the 2^-ΔCT ^index is several times higher for miR-BART7-3p than for other BART miRNAs. These data are representative of two similar experiments.

Our NPC xenografts are propagated in nude mice by sub-cutaneous inoculation of small tumor fragments which grow subcutaneously without invasion of underlying organs and tissues and therefore are well tolerated. We could collect plasma samples from mice carrying relatively large NPC xenografts (C15, C666-1 and C17) and also from mice carrying a xenografted EBV-negative human epithelial tumor (CAPI) used as a negative control [21]. The average ratio of tumor to mouse body mass was about 6 to 8%. Samples from 3 or 4 mice carrying the same xenografted tumor line were pooled and assessed for EBV DNA load. High DNA copy numbers were obtained for C15, C666-1 and C17 but not CAPI mice (Table 1). Total RNA was extracted from 100 μl of each plasma pool and subjected to multiplexed RT for the panel of miR-BART 1-5p, 5, 7-3p, 12 and 13 followed by single mode real-time PCR. The cellular microRNA miR-146a which is known to be abundant in blood plasma was used as an endogenous reference [23]. As shown in Figure 4 and Table 1, the most abundant BART miRNAs were found in plasma samples from mice carrying the C15 or C666-1 NPC tumors, consistent with the relative abundance of these microRNAs in the corresponding xenografted tumors. In contrast, low amounts of BART miRNAs were found in plasmas from mice carrying the C17 NPC. The miR-BART 7-3p was the most abundant in all cases. In contrast, the 2^-ΔCT ^was very low for miR-BART1-5p. There was no miR-BART detection in the pool of plasma samples from CAPI mice.

**Table 1:** Detection of BART miRNAs in plasma samples from xenografted mice

		C15	C666-1	C17	CAPI
**Tumor mass/Body mass (average ratio)**		6%-8%	6%-8%	6%-8%	6%-8%
**Plasma DNA viral load (copies/ml)**		6298	6298	50989	< 200
**2^-ΔCt^**	**ebv-miR-BART5**	1.516	1.542	< 10^-4^	< 10^-4^
	**ebv-miR-BART7-3p**	13.017	16.66	0.173	0.009
	**ebv-miR-BART13**	2.329	1.79	0.555	0.001

### Detection of BART miRNAs in the plasma of NPC patients

**Figure 5:**
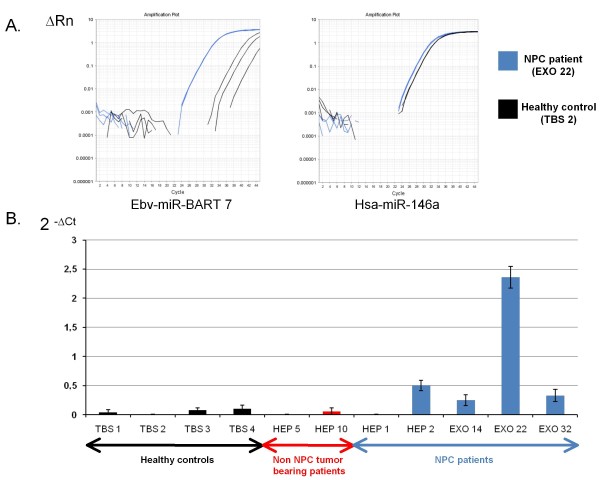
**Detection of BART miRNAs in plasmas samples from NPC patients**. Presence of ebv-miR-BART7-3p in human plasma samples was assessed by single-mode RT and real time PCR. Clinical and biological characteristics of plasma donors are summarized in Table 2. All five NPC patients had positive EBER-staining on tissue sections from their tumors. Two control patients were bearing non-NPC epithelial tumors: HEP5 (adenocarcinoma of unknown primary) and HEP10 (laryngeal squamous cell carcinoma). Three healthy donors (TBS 1, 2 and 3) were adult EBV-carriers as shown by serological investigations (detection of anti-VCA and -EBNA antibodies). The fourth healthy donor (TBS 4) was an EBV sero-negative adult. **Upper panel: **example of amplification plots of miR-BART 7-3p and mir-146a for one NPC patient (EXO 22) and one control subject (TBS 2). ΔRn stands for the magnitude of the fluorescence signal generated during the PCR at each time point (with background correction). **Lower panel: **histogram presenting the 2^-ΔCT ^values for miR-BART7-3p in the various human plasma samples. These data are representative of two similar experiments. Overall the differences between NPC patients and controls are statistically significant (p = 0.026 by the Mann Whitney test).

To demonstrate that the data obtained in our murine NPC model were relevant to human pathology we investigated the dissemination of the miR-BART 7-3p in plasma samples obtained from five consecutive NPC patients prior to any treatment. We used single-mode RT combined to real-time PCR. Plasma from three healthy EBV-carriers, a healthy donor not infected by EBV and two patients bearing non-NPC tumors were used as controls (Table 2). For each plasma sample, RNA was extracted from a total volume of 100 μl. The cellular miR-146a was used as an endogenous reference [23]. As shown in Figure 5, miR-BART7-3p was detected in the plasma samples from NPC patients at much higher levels than in samples from control donors, except for one of them (HEP 1). Overall the difference was statistically significant (p = 0.026 using the Mann-Whitney test).

**Table 2:** Clinical and biological characteristics of human subjects investigated for detection of plasma BART miRNAs

	Patient code	Age-sex- Country of origin	Tumor histological type (1)	Clinical Staging (2)	EBV status		Plasma viral DNA load (copies/ml) (3)	Ebv-miR-BART 7-3p 2 ^-ΔCt ^X1000
					EBER detection on tumor sections (3)	EBV serology Positive if > 0.2 Negative if < 0.1 (3)		
NPC PATIENTS	EXO 14	52-M-Vietnam	Non-keratinizing Undifferentiated	T3N3M1	EBER+	Not tested	4202	**250,5**
	EXO 22	51-M-France	Non-keratinizing undifferentiated	T3N2M1	EBER +	Not tested	1142	**2360,3**
	HEP 1	45-M-Cambodia	Non-keratinizing undifferentiated	T1N2M0	EBER +	Not tested	< 200	**6**
	EXO 32	40-F-Madagascar	Non-keratinizing undifferentiated	T3N2M0	EBER+	Not tested	< 200	**329,9**
	HEP 2	58-M-France	Non-keratinizing undifferntiated	T3N1M0	EBER+	Not tested	1589	**502,1**
NON-NPC TUMOR CARRIERS	HEP 5	69-M- France	Adenocarcinoma Multiple bone metastases of unknown primary	Not Applicable (NA)	NA	Anti-EBNA: 0,41 Anti-VCA: 4,08	< 200	**3,47**
	HEP 10	63-M-France	Larynx squamous cell carcinoma	T4N2M0	NA	Anti-EBNA:7,13 Anti-VCA: 3,73	< 200	**57,5**
HEALTHY CONTROLS	TBS 1	53-M-Algeria	NA	NA	NA	Anti-EBNA: 2,79 Anti-VCA: 2,46	< 200	**37,7**
	TBS 2	34-F-France	NA	NA	NA	Anti-EBNA: 0,07 Anti-VCA: 4,57	< 200	**3,47**
	TBS 3	29-F-France	NA	NA	NA	Anti-EBNA: 5,56 Anti-VCA: 1,65	< 200	**79,8**
	TBS 4	25-M-France	NA	NA	NA	Anti-EBNA: 0,05 Anti-VCA: 0	< 200	**99**

(1) WHO histological classification (2) according to ESMO guidelines (reference 31) (3) See Materials and Methods

## Discussion

In this study, we intended to investigate whether BART miRNAs are released in the extra-cellular medium by NPC cells and whether they are transported from the tumor site to circulating blood. Our data provide clear evidence that several BART miRNAs are secreted by C15 NPC cells *in vitro *in association with exosomes (Figure 3). Investigations of plasma samples in xenografted mice demonstrate that extra-cellular release of BART miRNAs also occurs *in vivo *and support the idea that they have enough stability and mobility to reach circulating blood (Figure 4). The data obtained from plasma samples collected in NPC patients are consistent with this conclusion (Figure 5).

Our study did not primarily intend to make quantification of BART miRNAs in various tumor backgrounds, however our results suggest that there are wide variations in the relative amounts of these microRNAs in NPC tumor lines. Except for miR-BART12, the highest concentrations of BART miRNAs were found in the C15 tumor with a slightly lower level in C666-1 and a much lower level in the C17 xenograft. These results are consistent with previous reports dealing with BART miRNAs or their precursors [14,24]. The low amount of BART miRNAs in the C17 xenograft might be related to its low number of EBV genome (about 2 copies per cell)[25]. However, according to Pratt et al. (2009) the amount of BART miRNAs is rarely correlated to the number of viral templates in latently EBV-infected cells [17]. In contrast with the Daudi lymphoid cell line, the NAD + C15 LCL - which is latently infected by an EBV isolate derived from the C15 tumor - also has substantial expression of the BART miRNAs with a profile somehow similar to the profile of C15. This could suggest that the viral genotype is more important than the cell background to determine the extent of BART miRNA expression.

Regardless of the RNA source, mir-BART7-3p consistently had the highest relative concentration among the 5 BART miRNAs of our panel. This confirms data reported by Pratt et al. [17]. This quantitative difference was even more marked in exosomes than in tumor RNAs, suggesting that miR-BART7-3p is produced at a higher level or is more stable than other BART miRNAs and possibly more efficiently packaged into exosomes.

In terms of diagnosis and patient monitoring, plasma BART miRNAs might become an interesting source of novel biomarkers. High concentrations of miR BART7-3p were detected in plasma samples from xenografted mice for 2 out of 3 NPC tumor lines as well as in plasma samples from 4 out of 5 NPC patients (Tables 1 and 2). We can only speculate about the absence or low level of miR-BART7-3p in the plasma of the NPC patient HEP 1. It might be the consequence of a relatively low tumor mass. It is noteworthy that a significant level of miR-BART7 was detected in the plasma from one NPC patient (EXO 32) in the absence of detectable EBV DNA in the same sample. This suggests that concomitant exploration of plasma EBV DNA and BART miRNAs will have the potential to provide distinct and complementary information about the tumor phenotype.

Additional investigations will be required on patient plasma samples - both NPC and controls - in order to address 2 questions: 1) Are concentrations of BART miRNAs consistently greater in the plasma of NPC patients by comparison with healthy carriers and patients bearing non-NPC tumors ? 2) Under which form, the BART miRNAs are transported in the plasma of NPC patients. Regarding this last question, recent publications suggest that there are two major modes of transport for extra-cellular microRNAs: either in a soluble form linked to proteins or packaged in nanoparticules, especially exosomes [10]. Some of our preliminary data are in favour of plasma BART miRNAs existing under both forms, a point that will deserve further investigations on a larger group of patients.

In terms of physiopathology, the finding of stable extra-cellular BART miRNAs suggests that they can play a role in cell-cell communications, for example communications between malignant and stromal cells. Horizontal transfers of microRNAs with impact on gene expression in recipient cells has already been demonstrated *in vitro *[6-8]. Exploring *in vivo *transfer of BART miRNAs to stromal cells will probably require investigations on tumor tissue sections [26]. If the hypothesis of microRNA horizontal transfers *in vivo *is confirmed, it will have important implications for our understanding of stromal proliferation, angiogenesis, immune escape and possibly metastatic processes. Elucidation of the cellular targets of BART miRNAs will be important in this respect. The pro-apoptotic gene encoding the PUMA protein has been identified as a target for miR-BART5; other cellular genes down-regulated by BART miRNAs will be probably identified in a near future [27].

## Conclusion

This study provides the proof of principle that the BART miRNAs are secreted by NPC cells *in vitro *and *in vivo *and can diffuse from the tumor site to the blood stream. It provides the rationale and some methodological clues for comparative detection and quantification of plasma BART miRNAs in series of NPC patients and control individuals.

## Methods

### Tumor xenografts and cell lines

C15 and C17 are xenografted EBV-positive NPC tumor lines permanently propagated by subcutaneous passage into nude mice [25]. Suspensions of NPC cells were obtained by dispersion of xenografted tumors using type II collagenase, sometimes combined with trypsin pre-treatment, as previously described [28]. C666-1 is an EBV-positive NPC tumor line which has been adapted to *in vitro *culture [29]. It was grown in RPMI supplemented with 25 mM Hepes and 7.5% FCS. Alternatively C666-1 cells were injected sub-cutaneously into nude mice for obtention of xenografted tumors (3 million cells mixed with 100 μl culture medium and 100 μl Matrigel, BD Biosciences, Le Pont-de-Claix, France). All experiments on xenografted NPC tumors were conducted in the animal facility of the Institut de Cancérologie Gustave Roussy, according to institutional guidelines. Daudi is an EBV-positive Burkitt lymphoma cell line [4]. NAD+C15 is a lymphoblastoid cell line (LCL) resulting from transformation of B lymphocytes from a normal adult donor by the C15 EBV-strain [19]. Daudi and NAD+ C15 were grown in RPMI supplemented with 10% FCS. The HeLa cervix carcinoma cell line was cultured in DMEM with 5% FCS.

### *In vitro *production of conditioned culture media containing exosomes

Cells of various types were incubated at appropriate concentrations in culture medium supplemented with 1.5% fetal calf serum (FCS) for 48 h, at 37°C under 5% CO2. C15 and C17 NPC cells were obtained by dispersion of xenografted tumors and incubated in 24 well plates at 1.2 million cells/well in 1.5 ml RPMI medium. HeLa cells were grown to 70% confluency in 175 cm^2 ^flasks and then incubated in 20 ml culture medium (DMEM). Daudi and NAD+C15 cells were incubated at 1 million cells/ml in RPMI (100 million cells/100 ml culture medium/175 cm^2 ^flasks). Following collection, conditioned media were clarified by centrifugation at 300 g for 10 min and at 1890 g for 15 minutes at 4°C to remove biggest cell remnants and debris and frozen at - 80°C.

### Purification of exosomes from culture media using a sucrose gradient

This procedure was adapted from the method described by Lamparski et al. [30]. All steps were performed at 4°C. Thawed conditioned culture supernatants (at least 400 ml) were first clarified by a centrifugation at 12 000 g for 35 min and then subjected to ultracentrifugation at 66 000 g for 2 h using a Ti45 Beckman rotor, resulting in a pellet designated as "nano-material pellet". Exosomes contained in this pellet were further purified by flotation on a cushion made of a sucrose solution in deuterium oxide (D_2_O). Practically, the nano-material pellet was redissolved in filtrated PBS (2 × 9 ml for an initial volume of 400 ml supernatant). One ml of sucrose/D_2_O solution (20 mM Tris/30% sucrose/D_2_O pH 7.4) was layed down carefully under 9 ml of nano-material solution at the bottom of a SW41 Ti polycarbonate tube. This two phase discontinuous gradient was subjected to ultracentrifugation at 76 000 g for 75 min on a SW41 Ti Beckman rotor. The faint band containing the exosomes at the surface of the cushion was then collected without disturbing the pellet. The exosomes were diluted 1:5 in PBS and pelleted by ultracentrifugation at 110 000 g in a SW41 Ti rotor for 90 min. Two additional washing steps were performed in a smaller volume (ultracentrifugation at 110 000 g using a TLA100.3 Beckman rotor). Washed exosomes were then processed for protein or RNA extraction. Exosome proteins were extracted in 20 μl of RIPA buffer (150 mM NaCl 5M, 50 mM Tris HCl pH:7,4, 5 mM EDTA, 0,1% SDS, 0,5% NaDOC, 0,5% NP40) supplemented with Complete anti-proteases (Roche, Basel, Switzerland). RNA extraction was started by solubilization in 800 μl of TRI REAGENT (Molecular Research Center, Cincinnati, OH).

### Transmission Electron Microscopy (TEM)

For negative staining, exosome fractions were observed after dilution in salt buffer (Tris 10 mM, pH 7.5, NaCl 150). Five microliters of solution was adsorbed onto a 300 mesh copper grid coated with a collodion film covered by a thin carbon film, activated by glow-discharge. After 1 min, grids were washed with aqueous 2% (w/vol) uranyl acetate (Merck, France) and then dried with ashless filter paper (VWR, France). TEM observations were carried out on a Zeiss 912AB transmission electron microscope in filtered low loss mode. Electron micrographs were obtained using a ProScan 1024 HSC digital camera and Soft Imaging Software system.

### Exosome characterization by western-blot

Exosome lysates were clarified by centrifugation at 16 000 g for 15 minutes at 4°C. Protein concentrations were determined using the Bradford protein Assay (Biorad Laboratories, Gif-sur-Yvette, France). The protein extracts (12.3 μg) were loaded on a Nupage Bis Tris MiniGel (Invitrogen, Carlsbad, New-Mexico) and migration was performed in non-reducing conditions. Monoclonal antibody against CD63 (TS63) was previously described (Charrin, Rubinstein at al, 2001). The gp96 cytoplasmic protein was detected with a rat monoclonal antibody (Stressgen, Ann Harbor, MI) and β-actin was visualized using a monoclonal antibody (AC-74) from Sigma Aldrich (St. Louis, MO).

### Collection, separation and storage of mouse and human plasma samples

Blood samples were collected from mice carrying xenografted NPC tumors under anesthesia by intra-cardiac puncture in EDTA tubes. Eight human plasma samples were collected after signature of informed consent from patients of the Institut de Cancérologie Gustave Roussy or Paris hospitals working in collaboration with this institute (Table 2). Five of these samples were collected from NPC patients prior to any treatment whereas two control samples were obtained from patients bearing non-NPC tumors (one adenocarcinoma of unknown primary and one larynx squamous cell carcinoma). Tumor staging was done according to ESMO (European Society of Medical Oncology) guidelines [31]. Additional control plasma samples were obtained from four healthy donors including three EBV-carriers and one EBV-sero-negative adult. Plasma was separated from blood cells by centrifugation at 1700 g at 20°C for 15 min and frozen at - 80°C.

### Assessment of EBV-status in tumor biopsies and in plasma samples

EBERs (Epstein-Barr encoded RNAs) which are small untranslated RNAs from EBV - totally distinct from the viral microRNAs and generally very abundant in NPC cells - were detected on tissue sections from the tumor biopsies by *in situ *hybridization using commercial kits, mainly from Ventana Medical System (Illkirch, France) [2]. Circulating antibodies directed to VCA (viral capsid antigen) and EBNA (Epstein-Barr nuclear antigen) were assessed in human plasma samples using the Vidas(r) EBV kit from Biomerieux (Lyon, France). EBV viral load in plasma samples was quantified as previously described [32]. Briefly: total DNA was extracted from 200 μl plasma aliquots using the QIAmp blood kit (Qiagen Inc., Courtaboeuf, France). Viral load was then determined by real-time quantitative PCR with primers designed to amplify the thymidine kinase gene of EBV (BXLF1). The copy number was determined by reference to a standard curve based on a tenfold serial dilution of a plasmid containing a unique copy of the BXLF1 genomic segment.

### RNA extraction from plasma samples

A variant of the TRIzol method was used to purify total RNA from cells as well as from exosomes produced *in vitro *according to the manufacturer instructions (TriReagent, Molecular Research Center, Cincinnati, OH). Total RNA from mouse and human plasma samples was extracted using the miRVana miRNA Isolation Kit (Ambion, Austin TX). Plasma was thawed on ice and 100 μl was mixed with 700 μl of Lysis/Binding buffer and incubated at room temperature for 5 min. RNA was then purified according to the manufacturer protocol except that centrifugation was extended to 15 min following acid-phenol/chloroform extraction. RNA was eluted in 100 μl RNAse free water. Finally RNA was quantified using a NanoDrop 1000 spectrophotometer.

### Single-mode reverse transcription and real time PCR amplification of EBV BART miRNAs

Detection of BART miRNAs was performed using reagents and protocols of the TaqMan MicroRNA Reverse Transcription and TaqMan MicroRNA Assay kits (Applied Biosystems, Foster City, CA). In this experimental system, reverse transcription (RT) is primed using a stem-loop primer specific of each microRNA. Each stem-loop primer has a specific linear portion complementary of the 3' end of the target microRNA and a loop portion containing a universal invariant target sequence. This RT results in a c-DNA joining the microRNA complementary sequence to the invariant sequence. This c-DNA is amplified by TaqMan PCR using a specific forward primer and a universal reverse primer in the presence of a specific hydrolysis probe. Due to spatial constraint of the stem-loop structure, this system is about 100 times more efficient at amplification of mature microRNAs than their precursors [33]. Reverse transcription was done in 15 μl reaction mix including 90 ng total RNA for cells and exosomes or 9.16 μl of the eluted RNA for plasma samples, 3 μl of the RT primer solution (final concentration: 50 nM), 0.15 μl dNTP (1 mM), 1 μl Multiscribe Reverse transcriptase (3.33 U/μl), 1.50 μl of 10× Buffer, 0.19 μl RNase inhibitor (0.25 U/μl) and nuclease free water. The reaction mix was incubated at 16°C for 30 min, 42°c for 30 min, 85°C for 5 min then frozen at -20°C. Single-mode real-time PCR was performed in a 20 μl reaction volume, containing 1.33 μl RT reaction mix providing the cDNA template, 1 μl of the primer mix including - for a given microRNA - the universal reverse primer (0.7 μM), the specific primer (1.5 μM) and the hydrolysis probe (0.2 μM) (TaqMan MicroRNA Assays, Applied Biosystems, foster City, CA), 10 μl of Fast Start Universal Probe Master mix (Roche, Basel, Switzerland) and RNase-free water. The first cycle included one step of 2 min at 50°C and one step of 10 minutes at 95°C. It was followed by 45 cycles including one step of 15 sec at 95°C and one step of 60 sec at 60°C. The following sets of primers and probes were purchased from Applied Biosystems (TaqMan MicroRNA assays): ebv-miR-BART 1-5p (197199_mat), ebv-miR-BART5 (197237_mat), ebv-miR-BART7-3p (197206), ebv-miR-BART12 (005725), ebv-miR-BART13 (005446), RNU44 (001094), hsa-miR-146a (000468), hsa-miR-21(000397). Amplification reactions were performed in an Applied Biosystems Step One Detection System. Data from RT-Q-PCR were analysed using the comparative C_T _method with RNU44, hsa-miR-21, hsa-miR-146a as endogenous references for tumor samples, exosomes and plasma samples, respectively. The 2 ^-ΔCT ^parameter was used as the index of target microRNA relative concentrations.

### Multiplex reverse transcription and real time PCR amplification of BART miRNAs

Detection of EBV-miR-BART 1-5p, 5, 7-3p, 12 and 13 was also performed in a multiplex mode, combining a multiplex Reverse Transcription (RT) stage and a stage of single PCR as recommended by the manufacturer. For this aim, a pool of RT stem-loop primers was made by mixing 6.25 pmoles of each primer. Practically, 25 μl of each primer solution were loaded in a 1.5 ml microtube and dried in a speed vacuum for 1 hour at 50°C. All RT dried primers were then solubilised in 100 μl of RNase free water. The same Taqman MicroRNA reverse Transcription kit used for single RT was used for multiplex with a few modifications: 90 ng input RNA was mixed with 4 μl of the RT primer mix (final concentration: 12.5 nM), 0.4 μl dNTPs (2 mM), 2 μl Multiscribe Reverse Transcriptase (5U/μl), 2 μl 10× RT Buffer, 0.25 μl RNase Inhibitor (0.25U/μl) and nuclease free water to reach a volume of 20 μl. Reaction parameters were identical to those used for single reverse transcription. The resulting cDNA was diluted by adding 180 μl of RNase-free water to the 20 μl reaction mix and stored at -80°C. Subsequent real time PCR was performed in the same conditions as when it was combined to single-mode RT, except that 9 μl of final RT reaction mix was mixed with other PCR reagents instead of 1.33 μl.

## Competing interests

The authors declare that they have no competing interests.

## Authors' contributions

CG and IB made RNA and c-DNA preparations and PCR analyses. CG was involved in the design of the study and preparation of the manuscript. AG and ASJ participated in exosome purification and study coordination. JK shared her expertise for handling of human plasma samples. BV, JG, PL and ST participated in the collection of clinical samples. VS and CA have assayed viral DNA load in plasma samples and assessed anti-VCA and -EBNA antibodies. SB has done electron microscopy of exosomes. PB participated in the design of the study and its coordination and drafted the manuscript. All authors read and approved the final manuscript.
